# Nasopharyngeal and temporal bone abscess following necrotizing otitis externa: a case report

**DOI:** 10.1093/jscr/rjae565

**Published:** 2024-09-05

**Authors:** Ahmad S Alharthi, Zohour A Almalki, Johara A Alnafie, Hazem K Althobaiti, Mohamed M Ibrahim

**Affiliations:** Department of Otolaryngology-Head & Neck Surgery, Alhada Armed Forces Hospital, Alhada, Taif City 26792, Saudi Arabia; College of Medicine and Surgery, Taif University, Alhawiyah, Taif City 26516, Saudi Arabia; Department of Otolaryngology-Head & Neck Surgery, King Abdullah Medical City, Muzdalifah road, Makkah City 24247, Saudi Arabia; Department of Otolaryngology-Head & Neck Surgery, Alhada Armed Forces Hospital, Alhada, Taif City 26792, Saudi Arabia; Department of Otolaryngology-Head & Neck Surgery, Alhada Armed Forces Hospital, Alhada, Taif City 26792, Saudi Arabia

**Keywords:** necrotizing otitis externa, Klebsiella pneumoniae, temporal bone abscess, otitis externa, nasopharyngeal abscess

## Abstract

Necrotizing otitis externa (NOE) is a severe infection primarily affecting the external auditory canal, seen mainly in immunocompromised individuals as patients with diabetes mellitus (DM). This case report highlights unusual complications of NOE: temporal bone and nasopharyngeal abscesses. These complications underscore the severity of NOE, particularly when caused by rare pathogens such as Klebsiella species. We detail the case of a 70-year-old male with uncontrolled DM who presented with severe right ear pain, purulent discharge, and significant postauricular swelling. Laboratory investigations revealed elevated inflammatory markers and poorly controlled diabetes. Cultures confirmed *Klebsiella pneumoniae*, and imaging showed diffuse edema and abscess formation in the temporal bone and nasopharynx. The patient was treated with intravenous Ceftazidime and ciprofloxacin for 6 weeks, followed by oral ciprofloxacin. Effective management of NOE necessitates a comprehensive, multidisciplinary approach. Early intervention, regular monitoring, and imaging are critical for promptly detecting and managing complications.

## Introduction

Necrotizing otitis externa (NOE) is a severe infection primarily affecting the external auditory canal, typically seen in immunocompromised individuals or those with underlying conditions [1]. *Pseudomonas aeruginosa* is the most commonly implicated pathogen. However, other organisms, including fungi and rarely identified bacteria like *Klebsiella pneumoniae*, can also cause this condition [2].

Patients often present with severe otalgia, otorrhea, and granulation tissue within the external auditory canal [3]. As the infection progresses, it can extend to adjacent structures, leading to cranial nerve palsies, skull base osteomyelitis, and potentially fatal intracranial complications [1]. Its progression to more complex complications involving adjacent structures, such as the nasopharynx and temporal bone, leading to abscess formation, which is rare but significantly morbid [4].

Individuals with compromised immunity, such as those with poorly controlled diabetes mellites (DM), HIV/AIDS, or receiving immunosuppressive therapy, are at higher risk [5]. Furthermore, anatomical variations or previous ear canal trauma can predispose individuals to NOE. Overuse or misuse of antibiotics can disrupt the natural balance of microbial flora, potentially creating an environment conducive to the overgrowth of opportunistic pathogens like *K. pneumoniae* [6].

This case report presents an instance of NOE complicated by both nasopharyngeal and temporal bone abscesses attributed to *K. pneumoniae* infection. We explore the clinical presentation, diagnostic challenges, management strategies, and the overall outcome in this context.

### Case presentation

A 70-year-old male, known for uncontrolled DM and hypertension (HTN), presented to the emergency room (ER) with a history of severe, persistent and unrelenting right ear pain started for three weeks, but it is increased in last few days accompanied by purulent discharge, significant postauricular swelling and headache. Notably the patient denied any subjective fever. Non-significant acute changes in hearing level, no vertigo. On initial assessment, the patient maintained stable vital signs and demonstrated an alert mental status. Cranial nerve examinations did not reveal any obvious deficits. Ear and otoscopic examination unveiled findings consistent with NOE, showing an edematous, erythematous external auditory canal with copious purulent discharge at the bony-cartilaginous junction with tragal tenderness, and notable postauricular swelling. Of clinical relevance, the patient had previously undergone multiple cauterizations for recurrent right aural polyps and was under regular follow-up with an ear, nose, and throat (ENT) specialist. Other ENT symptoms and examination was unremarkable.

Laboratory investigations revealed an elevated leukocyte count (13 150/μL), increased erythrocyte sedimentation rate (ESR; 62 mm/hr), markedly raised C-reactive protein (CRP; 52 mg/dL), and a significantly elevated glycated hemoglobin (HbA1c) level of 11.5%, indicative of poorly controlled diabetes. Peripheral blood culture yielded no growth, while the culture of the ear discharge confirmed the presence of *K. pneumoniae*. Notably, fungal staining and culture were negative for fungal elements.

**Figure 1:**
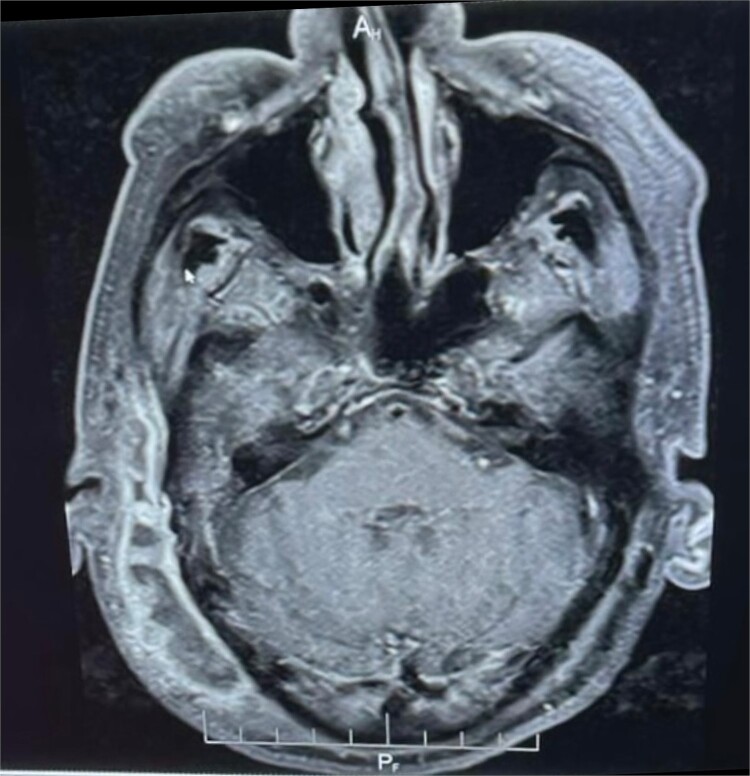
Enhanced MRI: Muitiloculated subcutaneous collection at the right temporoparietal region with marginal contrast enhancement, measuring ~0 cm x 2 cm. Marginally enhanced fluid collection on the right medial aspect of the nasopharynx.

Brain magnetic resonance imaging (MRI) demonstrated diffuse skin and subcutaneous tissue oedema involving the right temporoparietal and occipital regions, with multiloculated subcutaneous fluid collections displaying marginal contrast enhancement. Additionally, there was evident heterogeneous signal enhancement extending into the right external auditory canal, indicative of inflammatory changes. A smaller, marginally enhanced fluid collection was observed along the petrous apex in the right nasopharynx, suggestive of an abscess or localized infection (Fig. 1).

Following consultation with infectious disease (ID) specialists, the patient received intravenous Ceftazidime and ciprofloxacin for 6 weeks. Drainage of the postauricular swelling was performed with regular daily dressings. Collaborative efforts were initiated with the endocrine team to optimize glycemic control given the markedly elevated HbA1c. Patient was improved clinically and discharged on oral ciprofloxacin for additional four weeks.

Scheduled for regular follow-up appointments to monitor treatment response, glycemic control, and neurological status. Close collaboration between infectious disease, otolaryngology, and endocrinology teams, and regular imaging assessments to monitor the resolution of the infection and associated complications are integral components of the ongoing management plan.

## Discussion

A case report of NOE complicated by nasopharyngeal and temporal bone abscesses caused by *K. pneumoniae* infection was presented. The patient was a 70-year-old man with uncontrolled diabetes and HTN. This finding is consistent with the literature, which shows that diabetics account for 90% of cases [7, 8]. This was attributed to microangiopathy, hypoperfusion, and weakened immune and immune cell chemotaxis, which made the patient susceptible to infection [8].

The patients in this case had elevated leukocyte counts, ESR, CRP, and HbA1c levels. Similar laboratory findings were observed in NOE case reports [9]. NOE is typically diagnosed based on the clinical presentation and bacteriological findings. Imaging could play a supportive role [10].

The presence of *K. pneumoniae* was confirmed by culture of the current case’s ear discharge. *K. pneumoniae*, a gram-negative bacterium known for its aggressive infection potential, has been implicated as a causative agent in such cases only infrequently [11]. *K. pneumoniae* was linked to otogenic infections, particularly NOE, which resulted in abscesses in the nasopharynx and temporal bone in rare cases [12].

Our case’s MRI revealed diffuse skin and subcutaneous tissue oedema involving the right temporoparietal and occipital regions, as well as multiloculated subcutaneous fluid collections with marginal contrast enhancement. Furthermore, there was clearly heterogeneous signal enhancement extending into the right external auditory canal, indicating inflammatory changes. A smaller, marginally enhanced fluid collection was observed in the right nasopharynx along the petrous apex, indicating an abscess or localized infection. CT petrous temporal bone is useful in determining bone erosion in NOE, while MRI shows soft tissue extent, though these changes may be absent in early NOE [11]. The sensitivity of MRI for diabetic osteomyelitis is 90% and the specificity is 79% [11]. High-resolution imaging, such as MRI and computed tomography (CT), can help to determine the extent of inflammation in the petrous bone and surrounding structures. However, determining the response to treatment is difficult because inflammation-related tissue changes are visible by both modalities for a long time after the active inflammation has subsided [9]. Because there are no agreed-upon criteria for diagnosing NOE, clinical, radiological, and biological arguments must be used to maintain NOE diagnosis [13].

Ceftazidime and ciprofloxacin were prescribed for 6 weeks in the current case after multiple consultations. In addition, regular daily dressings were used to drain the postauricular swelling. Previous research indicated that ceftazidime with ciprofloxacin was the preferred antibiotic treatment. In most cases, this combination was prescribed empirically and was maintained when the germ isolated in the pus culture was sensitive to these antibiotics. When prescribed, the oral relay consisted primarily of ciprofloxacin [13]. Most gram-negative bacteria and methicillin-susceptible *Staphylococcus aureus* are covered by this medication regimen. These antibiotics are also widely distributed and highly concentrated in soft tissue, bone, and the central nervous system [8].

The endocrine team optimized the patient’s glycemic control, and the patient was scheduled for regular follow-up appointments to monitor treatment response, glycemic control, neurological status, and improvement. Although the severity of diabetes does not correlate with the extent of NOE disease, blood glucose management is critical [10]. For the current case, the collaborative effort of the infectious disease, otolaryngology, and endocrinology teams, as well as regular imaging assessments, were essential components of the ongoing management plan [8].

NOE can occasionally cause septic thrombosis of the lateral venous sinus, meningitis, other cranial nerve palsies, Bezold’s abscess, and intracranial extension manifesting as intracranial temporal or occipital abscesses (0.3%) [14], but it has a good prognosis when treated promptly. NOE surgical management was found to be indicated in aggressive or advanced disease with deep tissue involvement, facial nerve involvement, and refractory NOE despite 6 weeks of medical management [15].

## Conclusion

This case report highlights a rare and severe complication of necrotizing otitis externa caused by *K. pneumoniae* infection. Emphasizes the importance of a comprehensive, multidisciplinary approach in the treatment. Early detection and aggressive management of NOE will prevent its complications, which are associated with high mortality if left untreated.

## Conflict of interest statement

Authors have no conflict of interests.

## Funding

The work was not supported or funded by any drug company.
